# Psychological processes connecting childhood emotional abuse and healthy eating in university students

**DOI:** 10.3389/fpsyg.2026.1738521

**Published:** 2026-04-10

**Authors:** Chuqi Yan, Yanfei Wang, Zhiru Liang, Hongli Sun, Tiancheng Zhang, Bo Wang

**Affiliations:** 1 School of Sports Science, Jishou University, Jishou, China; 2 Department of Science and Education, Xilingol League Central Hospital, Xilinhot, Inner Mongolia, China; 3 Department of Hepatobiliary Surgery, Xilingol League Central Hospital, Xilinhot, Inner Mongolia, China; 4 Sanda University, Shanghai, China; 5 Clinical Medicine Major, Baotou Medical College, Inner Mongolia University of Science and Technology, Baotou, China

**Keywords:** childhood emotional abuse, depression, healthy eating, subjective wellbeing, university students

## Abstract

**Background:**

Childhood emotional abuse (CEA) has been associated with various psychological difficulties and health-related behaviors, yet its relationship with healthy eating among university students remains insufficiently explored. This study aimed to examine the association between childhood emotional abuse and healthy eating among university students and to further test the mediating roles of depression and subjective wellbeing (SWB).

**Methods:**

A cross-sectional survey design was employed, and participants were recruited using a convenience sampling method from several universities in China. A total of 3,007 university students participated. Measurement tools included the Childhood Emotional Abuse Scale, the Patient Health Questionnaire (PHQ-2) for depression, a single-item SWB scale, and the Healthy Eating Scale.

**Results:**

Correlational analyses showed that childhood emotional abuse was positively associated with depression and negatively associated with both SWB and healthy eating. Depression was negatively related to SWB and healthy eating, while SWB was positively related to healthy eating. Further chain mediation analyses indicated that depression and SWB partially mediated the association between childhood emotional abuse and healthy eating.

**Conclusion:**

These findings suggest that considering early adverse experiences and psychological states among university students may help explain the mechanisms underlying healthy eating behaviors. Interventions aimed at reducing depressive symptoms and enhancing SWB may contribute to promoting healthier eating behaviors among university students.

## 1 Introduction

Childhood emotional abuse (CEA) refers to a pattern of harmful behaviors by parents or caregivers, including verbal assaults, emotional neglect, control, humiliation, threats, or indifference. These behaviors can cause children to feel emotionally harmed, degraded, or unloved (Xu and Zheng, 2022). Unlike physical abuse, the emotional harm caused by such treatment may not immediately manifest, but its effects can be profound and long-lasting, potentially extending into adulthood. Previous studies have shown that CEA is associated with a variety of psychological and behavioral difficulties, including post-traumatic stress symptoms, depressive symptoms, reduced self-esteem, and impaired social functioning (Kwok et al., 2022; Thompson and Kaplan, 1996; Watts et al., 2021; Xu and Zheng, 2022). These long-term consequences highlight the importance of examining how early emotional adversity may influence health-related behaviors during emerging adulthood, particularly among university students who are undergoing critical developmental transitions. A meta-analysis including 29 studies with more than seven million participants reported a global prevalence of 36.3% for childhood emotional abuse (Stoltenborgh et al., 2012). Similarly, a meta-analysis of 32 studies found that the prevalence of CEA among Chinese university students was approximately 36.7% (Fu et al., 2018). Given the relatively high prevalence of CEA and its potential long-term consequences, understanding how early emotional adversity influences health behaviors among university students is an important issue for both psychological and public health research.

In recent years, researchers have increasingly explored the relationship between early adverse experiences and health behaviors, including eating patterns. Healthy eating refers to consuming nutritionally balanced foods that support physical functioning and long-term health, such as fruits, vegetables, and foods rich in vitamins, minerals, and dietary fiber, while limiting excessive intake of sugar-sweetened beverages and fast food (Lean, 2015). A growing body of research suggests that healthy eating plays an important role in promoting both physical and psychological wellbeing among university students (Wang et al., 2021; Muscaritoli, 2021). For instance, balanced dietary patterns have been associated with better mental health outcomes, improved cognitive functioning, and higher levels of life satisfaction in young adults. However, unhealthy dietary behaviors remain common among young people. For example, a large cross-national study including 222,401 adolescents from 61 countries reported that substantial proportions of adolescents had insufficient fruit and vegetable intake and frequent consumption of sugar-sweetened beverages and fast food (Shawon et al., 2023). For university students, the transition from adolescence to adulthood often involves increased autonomy in food choices, making this period particularly important for the development of long-term eating habits. Previous research suggests that early adverse experiences may influence eating behaviors through psychological and emotional processes. Individuals who experienced childhood abuse may have greater difficulty regulating emotions and coping with stress, which may increase the likelihood of maladaptive eating behaviors such as binge eating or excessive consumption of highly palatable foods (Dawson et al., 2022; Xiao et al., 2023). From an emotion regulation perspective, individuals exposed to emotional abuse often use food intake as a coping strategy to manage negative emotions, thereby increasing the likelihood of less healthy eating behaviors. Based on these findings, childhood emotional abuse may be negatively associated with healthy eating behaviors. Therefore, the present study proposes the following hypothesis: CEA is negatively associated with healthy eating (H1).

Persistent sadness, diminished interest, and reduced participation in daily activities are typical features of depressive symptoms reported among university students (Cui, 2015). According to a meta-analysis including 64 studies with 100,187 university students, the prevalence of depressive symptoms among university students was approximately 39.0% (Li et al., 2022). Previous studies have consistently shown that childhood emotional abuse is a significant risk factor for depression in adulthood (Young and Widom, 2014; Liu et al., 2024a; Liu et al., 2024b). Ecological systems theory suggests that individual development is shaped by interactions across multiple environmental contexts, including family relationships (Belsky, 1993). Within this framework, emotionally abusive family environments may disrupt emotional development and increase vulnerability to depressive symptoms (Cicchetti and Toth, 2016). Depressive symptoms may also influence health behaviors, including eating patterns. Individuals experiencing depression may engage in unhealthy eating behaviors as a coping strategy to alleviate negative emotions or psychological distress (Dakanalis et al., 2023). Previous studies have reported associations between depressive symptoms and unhealthy dietary choices, including increased consumption of sugary beverages and high-calorie foods (Zhang et al., 2019., Yan et al., 2026a). Therefore, depression may represent an important psychological mechanism linking childhood emotional abuse and unhealthy eating behaviors. Based on this reasoning, the present study proposes that depression mediates the relationship between CEA and healthy eating (H2).

In addition to depression, subjective wellbeing (SWB) represents another important psychological factor related to health behaviors. SWB refers to individuals’ overall evaluation of their life quality, including life satisfaction, positive emotions, and psychological fulfillment (Diener, 1984). According to attachment theory, early relationships with caregivers play a critical role in emotional development and the formation of psychological security (Zukauskiene, 2017). Experiences of emotional abuse may disrupt secure attachment patterns and lead to lower levels of SWB in later life. Self-determination theory further suggests that individuals with higher levels of wellbeing are more likely to engage in adaptive and health-promoting behaviors because their psychological needs for autonomy, competence, and relatedness are better satisfied (Ryan and Deci, 2000). Empirical studies have found that individuals with higher SWB tend to adopt healthier lifestyles, including healthier dietary habits (Ifroh et al., 2024). Therefore, SWB may represent another potential mechanism through which early emotional experiences influence eating behaviors. Accordingly, the present study proposes that SWB mediates the relationship between CEA and healthy eating (H3).

Furthermore, depression and SWB are closely related but conceptually distinct constructs. Depression reflects negative emotional states and psychological distress, whereas SWB represents a broader evaluation of life satisfaction and positive functioning. Previous research has shown that depressive symptoms can significantly reduce individuals’ SWB (Song et al., 2023; Yan et al., 2026b). From an emotion regulation perspective, persistent negative emotional states may undermine individuals’ ability to maintain positive evaluations of their life circumstances and psychological functioning (Gross, 2002). Therefore, it is possible that childhood emotional abuse first increases depressive symptoms, which subsequently reduces SWB, ultimately influencing health behaviors such as eating patterns. Based on this sequential psychological process, the present study proposes a chain mediation model in which depression and SWB sequentially mediate the relationship between CEA and healthy eating (H4).

**Figure 1:**
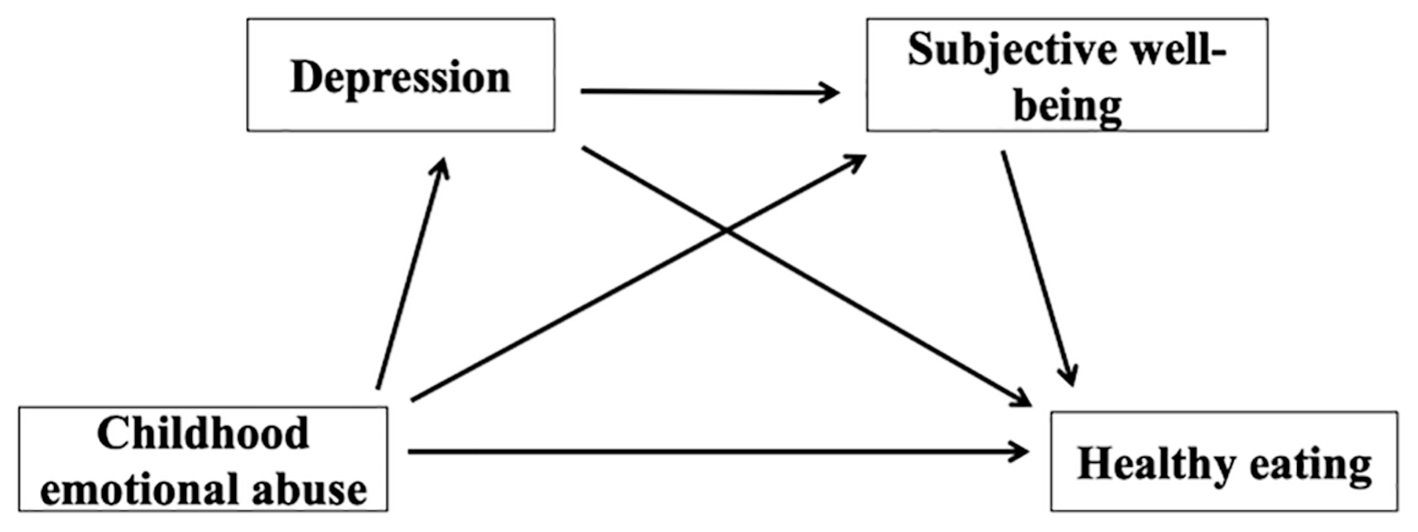
Hypothetical mediation model.

Although previous studies have examined the relationships among childhood emotional abuse, mental health, and health behaviors, few studies have simultaneously investigated the sequential mediating roles of depression and SWB in explaining how early emotional adversity may influence eating behaviors among university students. Addressing this gap, the present study aims to examine a chain mediation model linking childhood emotional abuse, depression, SWB, and healthy eating among university students (see Figure 1).

## 2 Methods

### 2.1 Participants

The survey was conducted in October 2024 across several universities located in Central, Northern, Northwestern, and Eastern regions of China. A convenience sampling approach was applied, with online questionnaires distributed through class group chats by teachers and counselors. Before proceeding, participants were required to read an informed consent form on the first page and provide agreement electronically. Eligibility criteria included: (1) being an undergraduate student, (2) aged between 16 and 24 years, and (3) voluntary completion of the survey. The selected age range reflects the typical age distribution of undergraduate students in China and corresponds to the developmental stage of emerging adulthood, during which health behaviors and psychological functioning undergo significant changes. Exclusion criteria were: (1) extremely short or excessively long response times, and (2) patterned or uniform responses indicating inattentive participation. To ensure data quality, responses with completion times shorter than one-third of the median survey completion time or longer than three times the median were removed. In addition, inattentive responding was identified through long-string analysis and low response variance across items. Participants who provided identical responses across multiple consecutive items were considered to exhibit patterned responding and were excluded from the dataset. Sample size was determined with reference to previous studies employing mediation analysis and was further constrained by available resources. A total of 3,146 questionnaires were initially collected. After applying the exclusion criteria and data cleaning procedures, 139 responses were removed due to invalid completion times or inattentive responding, resulting in a final analytic sample of 3,007 valid responses. The sample consisted of 1,267 males (42.1%) and 1740 females (57.9%), with a mean age of 19.03 ± 1.18 years. Of these, 596 (19.8%) were only children and 2,411 (80.2%) were non–only children. Regarding academic year, 1,371 (45.6%) were freshmen, 1,431 (47.6%) sophomores, 194 (6.5%) juniors, and 11 (0.4%) seniors. Maternal education levels were reported as primary school or below (29.3%), secondary school (55.2%), undergraduate (9.1%), and postgraduate or above (0.6%), with 5.9% unknown; paternal education levels were primary school or below (21.7%), secondary school (62.1%), undergraduate (10.1%), and postgraduate or above (0.8%), with 5.3% unknown.

### 2.2 Measurement tools

Considering the academic pressure faced by participants and the potential instability of online surveys, this study selected brief measurement tools to minimize disruption to participants’ daily learning and instructors’ teaching schedules. Using simplified measurement tools offers several advantages (Allen et al., 2022; Fisher et al., 2016), such as reducing cognitive and time burdens, minimizing response bias, enhancing participant engagement, and streamlining data processing and analysis. Brief screening instruments are widely used in large-scale epidemiological and behavioral research, particularly when the aim is to assess general psychological tendencies rather than provide clinical diagnoses. In the present study, the reliability and validity of the measurement tools were evaluated before hypothesis testing. Internal consistency reliability was assessed using Cronbach’s alpha coefficients, and construct validity was examined through correlation patterns among variables.

#### 2.2.1 Childhood emotional abuse

To assess the level of CEA in university students, this study employed the emotional abuse and emotional neglect subscales from the revised short form of the Childhood Trauma Questionnaire (Zhao et al., 2005). The scale consists of four items, with each subscale containing two questions. The items related to emotional neglect are reverse scored. A 5-point Likert scale was used for responses, ranging from 1 (never) to 5 (always). The total score ranges from 4 to 20, with higher scores indicating greater levels of abuse. The Kaiser-Meyer-Olkin (KMO) value was 0.772, indicating good sampling adequacy. In this study, the Cronbach’s *α* coefficients for the emotional abuse and emotional neglect subscales were 0.755 and 0.838, respectively. The overall Cronbach’s α coefficient for the CEA scale, composed of both subscales, was 0.845, indicating satisfactory internal consistency reliability.

#### 2.2.2 Depression

The Patient Health Questionnaire (PHQ-2) was used to assess the depression levels of university students (Levis et al., 2020; Liu et al., 2025). The scale consists of two items rated on a 4-point Likert scale, ranging from 1 (not at all) to 4 (almost every day). The total score ranges from 2 to 8, with higher scores indicating more severe depressive symptoms. The PHQ-2 is widely used as a brief screening tool for depressive symptoms in population-based studies and has demonstrated good psychometric properties in young adult samples. In this study sample, the Cronbach’s alpha coefficient for depression was 0.783.

#### 2.2.3 Subjective wellbeing

SWB was assessed using a single-item scale (Huppert et al., 2009) The question asked participants: “How would you rate your overall well-being during this period of your life?” Responses were rated on a 7-point scale, ranging from 1 (completely unhappy) to 7 (completely happy). Higher scores indicate stronger SWB experiences. Single-item measures of SWB have been widely used in large-scale surveys and have shown acceptable reliability and validity for assessing global life evaluation. Previous studies have demonstrated that such measures correlate strongly with multi-item well-being scales (Raudenská, 2023).

#### 2.2.4 Healthy eating

Healthy eating was assessed using three questions: “In the past week, how often did you (1) drink carbonated beverages? (2) eat fruit (excluding fruit juice)? (3) eat vegetables (including salads and non-fried potatoes)?” Response options ranged from 0 days to 5 or more days. The total healthy eating score was calculated by summing fruit and vegetable intake and subtracting carbonated beverage consumption, with higher scores indicating healthier eating habits (Add Health, 2016). This operationalization of healthy eating behaviors has been widely used in adolescent and university student health surveys and has shown acceptable reliability and construct validity (Waasdorp et al., 2019).

### 2.3 Covariates

Gender, age, father’s education level, mother’s education level, academic year, and only-child status were included as covariates in this study. These variables were included because previous studies have shown that demographic characteristics and family background factors may influence both psychological health and dietary behaviors among university students.

### 2.4 Statistical analysis

Descriptive statistics and correlation tests were first carried out using SPSS version 29.0. Prior to hypothesis testing, data were screened for normality and potential outliers. Skewness and kurtosis values were examined, and all variables fell within the acceptable range of ±2, indicating approximate normality. Multivariate outliers were evaluated using Mahalanobis distance. To examine the hypothesized chain mediation model, PROCESS macro (Model 6) was employed with 5,000 bootstrap iterations. In this model, CEA served as the predictor, healthy eating as the outcome, and depression together with SWB as sequential mediators. Bias-corrected bootstrapping was applied to generate 95% confidence intervals for the indirect pathways. The mediating role of depression via SWB was specifically evaluated. To address potential common method bias, Harman’s single-factor test was conducted. The first factor accounted for less than 40% of the total variance, indicating that common method bias was unlikely to substantially affect the results. Statistical significance was determined at the two-tailed level of *p <* 0.05.

## 3 Results

### 3.1 Common method bias test

Potential common method bias was examined using Harman’s single-factor procedure. The results of the unrotated factor analysis produced four factors with eigenvalues greater than one, with the largest factor explaining 20.438% of the variance. As this proportion falls far below the 40% reference value (Podsakoff et al., 2003), the influence of common method bias in the data is considered minimal.

### 3.2 Descriptive analysis

The results presented in Table 1 indicate significant gender differences in CEA (t = 3.36, *p <* 0.001) and healthy eating (t = −4.32, *p <* 0.001). Specifically, male participants reported higher levels of CEA compared to females, whereas female participants exhibited healthier eating habits than males. Significant differences across academic years were observed for CEA (*F* = 7.97, *p <* 0.001), depression (*F* = 5.91, *p <* 0.001), SWB (*F* = 3.83, *p <* 0.01), and healthy eating (*F* = 2.94, *p <* 0.05). Specifically, CEA scores were highest among fourth-year students, depression levels were highest among second-year students, SWB was highest among third-year students, and healthy eating habits were also highest among third-year students. Bonferroni post-hoc comparisons were conducted to further examine grade differences. The results indicated that freshmen reported significantly higher healthy eating scores than sophomores (*p* = 0.029). Similarly, freshmen reported significantly higher SWB than sophomores (*p* = 0.028). For depression, sophomores showed significantly higher scores than both freshmen (*p* = 0.003) and juniors (*p* = 0.014). In addition, sophomores reported significantly higher levels of childhood emotional abuse than freshmen (*p <* 0.001). No other significant grade differences were observed.

**Table 1:** Descriptive analysis of childhood emotional abuse, depression, subjective well-being, and healthy eating.

Variables		CEA		Depression		SWB		Healthy eating	
		Mean	SD	Mean	SD	Mean	SD	Mean	SD
Gender	Male	8.63	3.41	3.48	1.25	5.02	1.44	4.86	2.73
	Female	8.24	3.01	3.54	1.12	5.05	1.34	5.29	2.78
	*t*	3.36^***^		−1.46		−0.56		−4.32^***^	
Only child status	Only child	8.45	3.37	3.52	1.20	5.08	1.42	5.01	2.79
	Non-Only child	8.39	3.14	3.52	1.17	5.03	1.38	5.13	2.76
	*t*	0.39		0.05		0.73		−0.96	
Grade	Freshman	8.12	3.00	3.45	1.10	5.10	1.32	5.25	2.80
	Sophomore year	8.67	3.27	3.60	1.24	4.96	1.44	4.96	2.76
	Junior	8.32	3.65	3.33	1.27	5.21	1.4	5.28	2.56
	Senior	10.00	3.63	3.73	1.42	4.82	1.66	5.27	2.53
	F	7.97^***^		5.91^***^		3.83^**^		2.94^*^	

CEA, childhood emotional abuse; SD, standard deviation; Mean, mean value. *t*-values represent independent-samples t tests for gender and only-child status comparisons; F values represent one-way analysis of variance (ANOVA) for grade comparisons **p < 0.01; ***p < 0.001.

### 3.3 Correlation analysis

The results in Table 2 demonstrate significant associations among the study variables. Higher levels of CEA were accompanied by greater depression (r = 0.356, *p <* 0.001), whereas greater CEA corresponded to lower levels of SWB (r = −0.317, *p <* 0.001) and healthy eating (r = −0.217, *p <* 0.001). Increased depression was associated with reduced SWB (r = −0.386, *p <* 0.001) and poorer healthy eating (r = −0.210, *p <* 0.001). In contrast, higher SWB was linked to healthier eating behaviors (r = 0.185, *p <* 0.001).

**Table 2:** Pearson correlation matrix between relevant variables.

Variables	1	2	3	4
1. Childhood emotional abuse	–			
2. Depression	0.356^***^	–		
3. Subjective well-being	−0.317^***^	−0.386^***^	–	
4. Healthy eating	−0.217^***^	−0.210^***^	0.185^***^	–

***p <0.001.

### 3.4 Mediation model analysis

**Figure 2:**
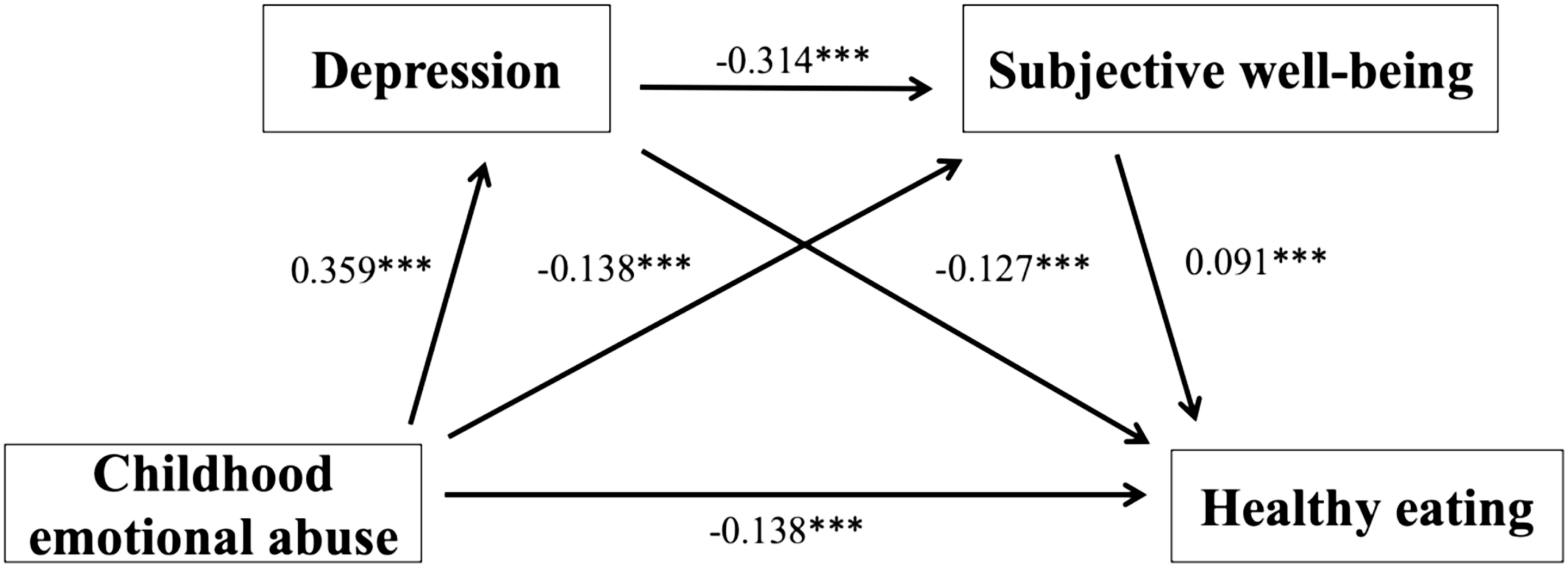
Chain mediation model of childhood emotional abuse and healthy eating in university students.

After controlling for demographic variables, the results presented in Table 3 and Figure 2 indicate that CEA was negatively associated with healthy eating behavior (*β* = −0.212, *p <* 0.001). This effect remained significant even after including the mediating variables (*β* = −0.138, *p <* 0.001). Specifically, CEA was positively associated with depression (*β* = 0.359, *p <* 0.001), and depression was negatively associated with healthy eating behavior (*β* = −0.127, *p <* 0.001). Additionally, CEA was negatively associated with SWB (*β* = −0.203, *p <* 0.001), while SWB was positively associated with healthy eating behavior (*β* = 0.091, *p <* 0.001). Finally, depression and SWB jointly played a significant mediating role in the relationship between CEA and healthy eating behavior (*β* = −0.314, *p <* 0.001).

**Table 3:** Mediation model test.

Outcome variables	Predictor variables	*β*	SE	*t*	*R*^2^	F
Healthy eating	CEA	−0.212	0.018	−11.844^***^	0.229	23.727^***^
Depression	CEA	0.359	0.017	20.902^***^	0.129	63.667^***^
SWB	CEA	−0.203	0.018	−11.426^***^	0.187	86.261 ^***^
	Depression	−0.314	0.018	−17.765 ^***^		
Healthy eating	CEA	−0.138	0.019	−7.129^***^	0.080	29.143***
	Depression	−0.127	0.020	−6.460^***^		
	SWB	0.091	0.019	4.700^***^		

Based on the results in Table 4, the total effect of CEA on university students’ healthy eating behavior was −0.212, with a direct effect of −0.138 and an indirect effect of −0.075, accounting for 35.37% of the total effect. This indicates that the mediating variables played a significant role.

**Table 4:** Mediation model path analysis of childhood emotional abuse and healthy eating in university students.

Path	Effect size	SE	Bootstrap 95% CI	Proportion of mediating effect
Total effect	−0.212	0.018	−0.247, −0.177	
Direct effect	−0.138	0.019	−0.175, −0.100	
Total indirect effect	−0.075	0.009	−0.092, −0.058	35.37%
CEA → Depression→ Healthy eating	−0.046	0.008	−0.061, −0.031	21.69%
CEA → SWB → Healthy eating	−0.019	0.004	−0.028, −0.010	8.96%
CEA → Depression → SWB → Healthy eating	−0.010	0.002	−0.015, −0.006	4.72%

## 4 Discussion

This study is a large-scale investigation into the relationships among CEA, depression, SWB, and healthy eating behaviors in university students. Additionally, the study aims to assess the mediating roles of depression and SWB in these relationships. The results indicate that CEA has both direct and indirect effects on healthy eating through its impact on depression and SWB. Furthermore, depression and SWB partially mediate the relationship between CEA and healthy eating. Specifically, CEA was significantly associated with higher levels of depression, higher depression was related to lower SWB, and lower SWB was further associated with lower levels of healthy eating.

The present study reveals that CEA is more prevalent among male students than female students, which aligns with previous research findings (Briere and Elliott, 2003). From a cultural perspective, this finding may also be influenced by traditional gender norms commonly observed in many Asian societies, including China. In such cultural contexts, males are often expected to demonstrate strength, emotional restraint, and independence, while the expression of vulnerability or psychological distress may be discouraged. Consequently, male students who have experienced emotional abuse may be less likely to disclose these experiences or seek psychological support. This cultural expectation of emotional suppression may increase the psychological burden associated with emotional abuse among men. Traditionally, societal expectations dictate that men should display strength, rationality, and emotional control. These cultural norms may discourage men from acknowledging or seeking help for emotional abuse. This is especially true in societies where expressing vulnerability or seeking support is seen as a sign of weakness or a threat to masculinity. Such cultural pressures could potentially intensify the psychological burden of emotional abuse on men, though they are less likely to express these difficulties openly (Cho et al., 2020). Regarding healthy eating behaviors, female students demonstrated significantly higher scores than males. Prior research has indicated that females are more likely to consume fruits, vegetables, and low-fat foods, whereas males tend to prefer high-calorie and fried foods (Herman and Polivy, 2008). These differences not only reflect variations in health awareness between genders but may also stem from the influence of gender-role socialization processes (Ahmadi, 2015). Additionally, cultural expectations regarding body image and health management may further reinforce these gender differences. In many East Asian cultural contexts, women are often encouraged to pay closer attention to dietary habits, body management, and health-related behaviors, which may contribute to the higher levels of healthy eating observed among female students. Differences in reported emotional abuse were also observed across academic years. Senior students (fourth-year) reported higher levels of emotional abuse, which may be attributed to more advanced cognitive maturity and greater capacity for self-reflection (Arnett, 2000). As graduation approaches, these students might be more inclined to recall childhood experiences and recognize instances of emotional harm that they were previously unaware of. In contrast, second-year students reported higher levels of depression, possibly reflecting a transitional stress period between the initial adjustment of the first year and the academic intensification of the third year. Increased academic workload, more difficult coursework, and uncertainty about the future may contribute to elevated psychological distress during this phase (Ramón-Arbués et al., 2020). Within the Chinese sociocultural context, academic achievement is often strongly emphasized by families and society, and university students may face considerable expectations regarding educational success and future employment. Such cultural emphasis on academic performance may further intensify psychological stress during critical academic periods. SWB and healthy eating behavior scores were relatively higher among third-year students. This may be due to greater adaptation to university life at this stage, along with improved time management and emotional regulation skills, which could foster a more positive psychological state and promote healthier lifestyle choices (Debra et al., 2024).

The present study found a negative association between CEA and healthy eating behaviors among university students, a finding consistent with prior research (Yuksel et al., 2020). On the one hand, previous studies have indicated that individuals who experienced CEA during childhood are more likely to exhibit unhealthy eating patterns and irregular eating behaviors in adulthood (Heim and Nemeroff, 2001). On the other hand, childhood trauma often impairs emotional regulation abilities, making individuals more likely to cope with negative emotions through eating, which may be linked to difficulties in emotional regulation and associated with behaviors like binge eating and emotional eating (van Strien, 2018). However, some studies have suggested that the relationship between childhood adversity and eating behaviors may be more complex and may vary depending on individual and contextual factors, such as coping strategies, social support, or cultural background (Li et al., 2024; Yoon and Johnston, 2025). Therefore, although the present findings support a negative association between CEA and healthy eating behaviors, the underlying mechanisms may involve multiple interacting psychological and environmental influences. The findings of the present study further support this association, suggesting that CEA may be related to healthy eating in adulthood, thus supporting Hypothesis 1. Nevertheless, given the cross-sectional nature of the current study, the direction of this relationship should be interpreted cautiously, and future longitudinal studies are needed to better clarify the causal pathways between childhood emotional abuse and eating behaviors.

This study identified depression as a significant mediator in the relationship between CEA and healthy eating behaviors, suggesting that depressive symptoms may serve as a key psychological mechanism through which early adverse experiences shape adult lifestyle choices. This finding aligns with previous research (Chang et al., 2021; Zhang et al., 2019; Yang et al., 2025). A recent meta-analysis of cohort studies reported that more than half of global depression cases may be attributable to self-reported childhood maltreatment. Notably, reducing the prevalence of childhood abuse by just 10 to 25% could potentially prevent 31.4 to 80.3 million cases of depression worldwide (Li et al., 2016). Additionally, evidence from 20 longitudinal and 21 cross-sectional studies consistently demonstrates that higher levels of depressive symptoms are associated with poorer eating behaviors (Lassale et al., 2019). Meta-analytic findings further indicate that eating interventions are a promising strategy for reducing both the risk and severity of depressive symptoms (‘An Anti-Inflammatory Diet as a Potential Intervention for Depressive Disorders’, 2019). In line with emotion regulation theory (Gross, 2002), CEA may be associated with difficulties in identifying and regulating emotions, which in turn may be linked to higher levels of depressive symptoms. In a depressed state, individuals are more likely to engage in emotional eating, particularly of energy-dense foods high in sugar and fat, as a maladaptive coping strategy to alleviate negative affect (Dakanalis et al., 2023). This behavioral pattern is associated with unhealthy eating habits. These findings provide empirical support for Hypothesis 2 of the current study.

This study found that SWB mediates the relationship between CEA and healthy eating behaviors among university students, consistent with previous findings (Ifroh et al., 2024; Xiao et al., 2023; Zukauskiene, 2017). Existing studies have reported that CEA is negatively associated with SWB (Kelifa et al., 2021). Cross-sectional evidence also suggests that individuals reporting lower levels of SWB tend to report less healthy lifestyle patterns, including aspects related to eating (Ifroh et al., 2024). In line with Self-Determination Theory (Ryan and Deci, 2000), when basic psychological needs—such as relatedness and autonomy—are not adequately met, individuals may experience diminished wellbeing and weakened intrinsic motivation to engage in healthy lifestyle behaviors. CEA may chronically undermine the fulfillment of these needs, thereby indirectly decreasing SWB and reducing one’s motivation and capacity to maintain healthy eating patterns. Furthermore, cross-sectional findings suggest that individuals reporting higher levels of SWB tend to report better self-monitoring of health behaviors, greater resistance to external temptations, and more health-conscious eating patterns, such as regular eating patterns and lower reported intake of high-calorie foods (Ifroh et al., 2024). The findings of this study provide further support for the above viewpoint, suggesting that SWB may be an important psychological mechanism linking CEA and Healthy eating, thereby supporting Hypothesis 3.

This study found that depression was partially associated with the relationship between CEA and SWB, and that SWB may be linked to healthy eating. This pathway is consistent with previous findings (Öztürk and Mutlu, 2010), and can be interpreted within the framework of social ecological theory (Crandon et al., 2022). This theory emphasizes that an individual’s mental health is influenced by multiple levels of factors, including individual, family, and societal contexts. In the pathways observed in the present study, CEA is associated with decreased SWB through increased levels of depression. This relationship may be influenced by various factors within the individual’s environment. For example, previous studies have shown that individuals who have experienced adverse childhood events are more likely to exhibit higher levels of depression (Briere and Elliott, 2003; Teicher and Samson, 2013). Individuals experiencing depression often report feelings of helplessness, self-devaluation, and persistent negative affect, which are commonly associated with lower levels of life satisfaction and SWB (Briere and Elliott, 2003). Within a social ecological framework, depression is closely associated with reduced SWB, which in turn is related to eating behaviors (Ford et al., 2014). SWB is a key indicator of psychological health. Individuals with higher levels of wellbeing are generally more capable of regulating their emotions and making health-oriented behavioral choices. As such, they are more likely to maintain balanced and nutritious eating patterns (Herman and Polivy, 2008). This study further reveals a potential pathway through which CEA may be associated with healthy eating behaviors via a chain mediation mechanism involving depression and SWB. This finding thus provides support for Hypothesis 4.

This study, grounded in theoretical frameworks from psychology and behavioral medicine, integrates socio-ecological theory and emotion regulation theory to examine the chain mediation mechanism through which CEA influences healthy eating in university students via depression and SWB. The findings reveal that CEA is associated with poorer healthy eating, higher levels of depression, and lower SWB among university students. This result enriches the application of emotion regulation theory in the field of eating behavior and provides further empirical evidence for understanding how early adverse experiences may influence health-related behaviors in young adulthood. The theoretical contributions of this study are twofold. First, it addresses a research gap by examining how childhood emotional abuse may influence university students’ healthy lifestyle through emotional variables, highlighting the mediating chain of depression–SWB as an important psychological pathway. Second, the results support the role of emotional factors in health behavior formation, suggesting that depression and SWB may serve as key psychological links between early emotional experiences and later lifestyle behaviors. In addition to its theoretical contributions, this study also has practical implications for university health promotion. Universities may consider integrating psychological support with health education programs to promote healthier eating behaviors. For example, interventions targeting depressive symptoms and enhancing SWB—such as cognitive–behavioral training, mindfulness programs, or positive psychology interventions—may indirectly support healthier lifestyle choices among students. Early psychological screening and counseling services may also help identify students who are at higher risk due to adverse childhood experiences. Future research should further explore additional factors that may influence these relationships, such as self-control, self-esteem, social support, and health beliefs. Longitudinal studies are also needed to clarify the temporal relationships among childhood emotional abuse, psychological factors, and health behaviors. Moreover, incorporating multiple data sources or objective indicators of dietary behavior may improve the reliability and ecological validity of future findings. In summary, this study provides empirical evidence for the psychological pathways linking childhood emotional abuse and healthy eating among university students and highlights the importance of considering psychological wellbeing when promoting healthy lifestyle behaviors in higher education settings.

Although the study offers strengths in sample size, pathway analysis, and methodological design, the findings should be interpreted with caution. First, the study employed a cross-sectional design, which, although capable of revealing significant associations among variables, precludes the inference of causal relationships. Future research is encouraged to adopt longitudinal or experimental designs to verify the temporal and causal nature of the proposed mediation pathways. Second, the use of convenience sampling—albeit involving multiple universities—may introduce selection bias and limit the generalizability of the findings to broader populations. Third, regarding measurement tools, SWB was assessed using a single-item indicator, which may not fully capture the complexity of individuals’ wellbeing experiences. In addition, because SWB was measured using a single-item indicator, it was not possible to conduct a formal discriminant validity test between SWB and depression. Although these constructs are conceptually distinct, this measurement approach may limit the ability to fully assess construct differentiation. Future research should employ multidimensional and validated measurement instruments to better distinguish between related psychological constructs. Similarly, healthy eating behavior was measured using only three self-reported items, focusing on a narrow set of behaviors and lacking coverage of key aspects such as eating frequency, food types, and nutritional balance. This limitation may have impacted the precision and validity of the measurement. Future studies should consider employing multidimensional and psychometrically validated instruments to improve measurement quality and enhance the robustness of research conclusions. Fourth, the present study included a limited number of control variables. Potentially important confounding factors, such as socioeconomic status, body mass index (BMI), and physical activity levels, were not included in the current analysis. These variables may influence both psychological states and health-related behaviors. Future research should incorporate these factors to improve the rigor and explanatory power of the analytical model. Finally, although Harman’s single-factor test was conducted to examine common method bias, reliance on a single statistical procedure may not completely eliminate the possibility of such bias. Future studies are encouraged to adopt additional techniques, such as marker variables or confirmatory factor analysis, to provide more robust control of common method variance. Moreover, the current model did not include potentially important variables such as social support, self-control, and health literacy. Expanding the conceptual framework to incorporate these factors could enrich our understanding of the mechanisms underlying university students’ health behaviors and provide a more comprehensive theoretical foundation for intervention development.

## 5 Conclusion

The present research indicates that CEA is closely linked to healthy eating behaviors in university students, with depression and SWB acting as partial mediators. These two psychological factors may function independently or together in a sequential pathway. However, the use of single-wave data prevents causal interpretation, and bidirectional relationships among the variables remain possible. The results emphasize the relevance of early emotional experiences in shaping dietary patterns in young adults and highlight the contribution of psychological conditions in this process. Addressing depressive symptoms and enhancing wellbeing could support healthier lifestyle practices and better behavioral outcomes. This work offers both a conceptual perspective and empirical insights that may inform health promotion and psychological counseling strategies in higher education.

## Data Availability

The raw data supporting the conclusions of this article will be made available by the authors, without undue reservation.
